# tDCS-Induced Memory Reconsolidation Effects: Analysis of Prominent Predicting Factors

**DOI:** 10.3389/fnins.2022.814003

**Published:** 2022-03-17

**Authors:** Maria Cotelli, Clarissa Ferrari, Elena Gobbi, Giuliano Binetti, Rosa Manenti, Marco Sandrini

**Affiliations:** ^1^Neuropsychology Unit, IRCCS Istituto Centro San Giovanni di Dio Fatebenefratelli, Brescia, Italy; ^2^Statistics Service, IRCCS Istituto Centro San Giovanni di Dio Fatebenefratelli, Brescia, Italy; ^3^MAC Memory Clinic and Molecular Markers Laboratory, IRCCS Istituto Centro San Giovanni di Dio Fatebenefratelli, Brescia, Italy; ^4^School of Psychology, University of Roehampton, London, United Kingdom

**Keywords:** healthy older adults, subjective memory complaints, mild cognitive impairment, memory, cognitive reserve

## Abstract

**Background:**

Memory impairment is among one of the greatest cognitive complaints in midlife and in old age. Considering the importance of good memory functioning in everyday life, it is crucial to study interventions that can reduce the natural decline in this cognitive function. Transcranial Magnetic Stimulation (TMS) studies have demonstrated that the lateral prefrontal cortex (PFC) plays a causal role in enhancing episodic memory recall through reconsolidation. Using a similar paradigm with transcranial direct current stimulation (tDCS) over the left lateral PFC, facilitation effects were observed in delayed memory retrieval in older adults with subjective memory complaints (SMCs) and amnestic Mild Cognitive Impairment (aMCI). However, it remains unclear which potential factors (i.e., tDCS group, cognitive reserve, education level, diagnosis and encoding performance) directly and/or indirectly modulate the tDCS-induced memory reconsolidation effects.

**Methods:**

We reanalyzed data acquired in our previous tDCS studies with 22 SMC and 18 aMCI participants from the perspective of predicting delayed memory retrieval performance. These studies included a learning session on Day 1, a reactivation by a contextual reminder followed by 15 min of tDCS session on Day 2 (24 h after Day 1), and two retrieval sessions (free recall and recognition) tested on Days 3 and 30 (48 h and 30 Days after Day 1).

**Results:**

Univariate models showed that tDCS group (sham vs. active) significantly predicted memory recognition (but not free recall), evidenced by higher scores in the active tDCS group than in sham group, confirming our previous results. Encoding performance and diagnosis (SMC vs. aMCI) significantly predicted memory retrieval, suggesting higher performances in individuals with SMC than in those with aMCI. Regarding cognitive reserve, higher leisure time activity subscores significantly predicted better memory recognition. Finally, multiple models did not show any tDCS group × predictor interaction effects, indicating that the effects of the predictors on retrieval occurred irrespective of tDCS group.

**Conclusion:**

Our results shed light on predicting factors of episodic memory retrieval in this reconsolidation paradigm in individuals with SMC and aMCI. The findings suggest that multifactorial interventions program may be most promising to slow cognitive decline and delay the onset of dementia.

## Introduction

The ability to store and remember information plays a fundamental role throughout the existence of an individual from the earliest stages of learning through aging. Episodic memory is responsible for remembering personally experienced events and shows the greatest age-related decline.

Memories are acquired (encoding), maintained (storage), and later retrieved (retrieval). Notably, after encoding, memories are unstable (fragile) and vulnerable to interference, but as time passes, memories stabilize or consolidate and become resistant to interference (McGaugh, 2000). In the first few hours after encoding, morphological changes in hippocampal pathways result in the first type of consolidation process (at the cellular level) leading to memory stabilization. Subsequently, a gradual reorganization of the neural networks (lasting hours to years, depending on the type of memory) induces further consolidation (Frankland and Bontempi, 2005; Dudai, 2012). Thereafter, consolidated memories can return to unstable (fragile) states if reactivated during retrieval or by a reminder cue (Dudai, 2012). Once reactivated, memory reconsolidation can act to restabilize the consolidated, existing memories (Sandrini et al., 2015). During a time-limited reconsolidation window, behavioral, pharmacological or non-invasive brain stimulation interventions can be used to weaken, strengthen or update consolidated memories (Sandrini et al., 2015, 2019; Lee et al., 2017; Elsey et al., 2018).

Memory performance may be facilitated through an interaction of tDCS with the mechanisms of consolidation or reconsolidation (Flöel et al., 2012; Sandrini et al., 2014, 2016, 2019; Manenti et al., 2017, 2020).

It has been proved that the presentation of cues related to previously encoding episodes during periods of awake rest or sleep generates patters of activity in the hippocampus that are consistent with the reactivation of neuronal memory, and strengthens subsequent episodic memory for these episodes (Rasch and Born, 2007; Tambini et al., 2018; Alm et al., 2019).

In this respect, a particularly interesting line of research involves studies that focus on the application of new procedures that can strengthen memory traces in healthy older adults and in subjects at risk of developing Alzheimer’s disease (AD), such as individuals with subjective memory complaints (SMC) and amnesic mild cognitive impairment (aMCI). An important contribution to this research comes from the introduction of non-invasive brain stimulation. Several studies have recently demonstrated the possibility of transcranial direct current stimulation (tDCS) to strengthen or enhance episodic memory in physiological and pathological aging (Sandrini et al., 2020; Huo et al., 2021).

We have demonstrated that repetitive Transcranial Magnetic Stimulation (rTMS) applied to the lateral prefrontal cortex (PFC), a brain region critically involved in episodic memory (Fletcher and Henson, 2001; Manenti et al., 2012; Sandrini et al., 2013), during reconsolidation enhanced episodic memory recall in young adults (Sandrini et al., 2013).

Using a similar paradigm with tDCS in healthy older adults, we have shown that active tDCS applied over the left lateral PFC during consolidation (Sandrini et al., 2019) or reconsolidation (Sandrini et al., 2014; Manenti et al., 2016) induced long-lasting enhancements in verbal episodic memory recall (up to 30 days).

Moreover, we have recently reported that the application of active tDCS over the left PFC during reconsolidation induced verbal episodic memory enhancement (i.e., recognition scores) in individuals with SMC (Manenti et al., 2017) and aMCI (Manenti et al., 2020).

Importantly, a recent study (Vaqué-Alcázar et al., 2021) replicated our findings (Manenti et al., 2017) in an independent sample, which confirmed that tDCS, through the modulation of memory reconsolidation, is capable of enhancing performance in people with SMC. This study also suggested that individuals with more preserved structural and functional integrity might benefit from this intervention.

There is also evidence that education moderates the effects of tDCS on memory performance in healthy aging individuals (Berryhill and Jones, 2012) and patients with aMCI or AD (Krebs et al., 2020). However, in this latter study, the directions of the education effects were inconsistent, suggesting additional influencing factors (Krebs et al., 2020).

Thus, it remains unclear which potential factors (i.e., cognitive reserve, education level, diagnosis, encoding performance) moderate tDCS-induced memory reconsolidation effects in populations at risk of AD.

A number of studies have demonstrated that cognitive reserve represents a protective factor in subjects at risk for cognitive decline (Stern, 2002, 2009; Whalley et al., 2004; Richards and Deary, 2005; Andel et al., 2006; Roe et al., 2007).

Cognitive reserve is a hypothetical construct that implies that people have different capacities to withstand changes. In situations often leading to functional decline, some subjects could resist associated changes and maintain their cognitive functioning (Stern, 2012). Cognitive reserve refers to individual differences in solving tasks despite similar degrees of destruction or degeneration such that there is a reserve against the pathology. Measures of cognitive reserve include socioeconomic status, education, occupational attainment, or participation in leisure activities.

To address this question, we reanalyzed data acquired in our previous studies with SMC and aMCI participants (Manenti et al., 2017, 2020) running a new analysis aimed to predict changes in memory performance induced by tDCS. In particular, the aim of this study was to investigate how the magnitude of tDCS effects might be directly and/or indirectly modulated by factors such as cognitive reserve, education level, diagnosis and encoding performance.

## Materials and Methods

### Procedure

Data from 22 individuals with SMC (age = 74.5 SD = 5.9 years) and 18 with aMCI (age = 75.3 SD = 3.7 years) were included in the analysis. All participants had normal or corrected-to-normal vision and were native Italian speakers. The SMC sample was defined as: (1) Mini Mental State Examination (MMSE) score between 27 and 30 (Folstein et al., 1975); (2) a score of more than 1.0 SD at Everyday Memory Questionnaire (EMQ) above the mean score obtained in a group of healthy older participants [mean 37.3, SD 8.4; (Manenti et al., 2016)]; (3) normal objective memory performance on neuropsychological tests; (4) normal objective cognitive performance in all the administered tests; (5) normal scores in functional assessment; (6) absence of mood and anxiety disorders; (7) absence of criteria for a diagnosis of dementia according to DSM-V (Regier et al., 2013).

The aMCI sample was defined as: (1) subjective memory complaints; (2) MMSE score between 24 and 30; (2) global Clinical Dementia Rating (CDR) score of 0.5; (3) predominant episodic impairment on a standard neuropsychological test (i.e., story recall; Rey Auditory Verbal Learning Test, recall; Rey-Osterrieth Complex Figure, recall); (4) preservation of functional activities; (5) absence of criteria for a diagnosis of dementia according to DSM-V (Regier et al., 2013); (6) absence of mood and anxiety disorders.

SMC and aMCI participants were randomized into two groups: (a) active tDCS (anode over the left lateral PFC and cathode over right supraorbital area) or (b) sham tDCS. Each participant was assigned to a tDCS group following stratified randomization based on Mini-Mental State Exam (MMSE) score and age. The participants and study team members did not know which tDCS condition was being applied at any point in the experiment. SMC and aMCI individuals underwent an extensive neuropsychological assessment. The battery took approximately 90 min and included Mini Mental State Examination (MMSE) (Folstein et al., 1975) for assessment of global cognition, Raven’s Colored Progressive Matrices for non-verbal reasoning, verbal fluency (phonemic and semantic) for language production, Token Test for language comprehension, Rey–Osterrieth Complex Figure (ROCF)-Copy for visuo-constructional abilities, Trail Making Test part A and part B for attention and executive function, Auditory Verbal Learning Test (AVLT), immediate and delayed recall, and Story Recall for verbal episodic memory, Rey–Osterrieth Complex Figure (ROCF)-Recall for non-verbal episodic memory and Digit Span for verbal short term memory (Lezak et al., 2004).

Functional abilities, memory complaints and symptoms of depression and anxiety were assessed. In addition, all participants were administered the Cognitive Reserve Index questionnaire (CRIq), which provides a standardized measure of the cognitive reserve accumulated by individuals through their lifespan. The CRIq includes demographic data and items grouped into three sections: education, working activity and leisure time, each of which results in a subscore that are combined to comprise the total score (Nucci et al., 2012).

### Transcranial Direct Current Stimulation

A tDCS stimulator (BrainStim, EMS, Bologna, Italy) delivered a constant low-intensity (1.5 mA) current for 15 min through two saline-soaked sponge electrodes (7 × 5 cm; current density: 0.043 mA/cm^2^) (Bikson et al., 2016; Antal et al., 2017). Active or sham stimulation mode was selected by entering blind codes so that the experimenter that applied the tDCS did not know which type of stimulation was being applied. The electrodes were secured using elastic bands, and an electroconductive gel was applied under the electrodes to reduce contact impedance (Manenti et al., 2013; Sandrini et al., 2014, 2016).

The anode electrode was placed over F3 (left lateral PFC) with the long side parallel to the sagittal line, and the cathode electrode was located over Fp2 (right supraorbital area, above the arcus superciliaris) with the long side parallel to the horizontal line (DaSilva et al., 2011) in accordance with the 10–20 EEG international system.

In the active tDCS condition, the current was applied for 15 min (with a ramping period of 10 s at the beginning and at the end of the tDCS session), whereas in the sham condition, the current was turned off 10 s after the beginning and was turned on for 10 s at the end of the stimulation period (Manenti et al., 2013). Immediately after the stimulation session, the subjects were required to report any perceptual sensations to verify the comparability of the tDCS sensations induced by active and sham stimulation conditions (Fertonani et al., 2015).

### Experimental Memory Task

**FIGURE 1:**
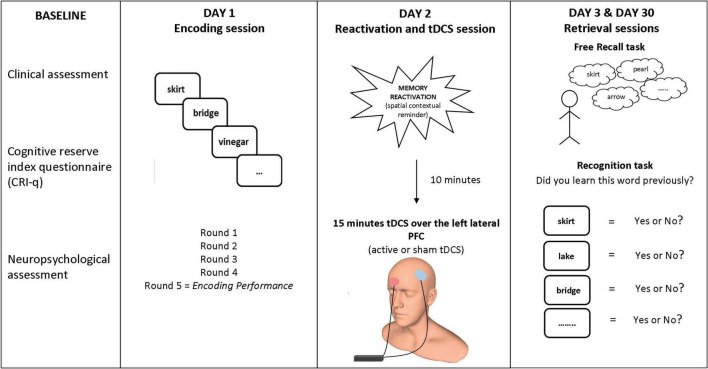
Experimental paradigm. Participants learned 20 words on Day 1. On Day 2 (24 h later), tDCS (active or sham) was applied over the left lateral PFC (anode over left PFC and cathode over the right supraorbital area) after a spatial contextual reminder. Memory retrieval (free recall and old/new recognition) was tested 48 h later (Day 3) and 30 days later (Day 30). Human head model from http://www.ir-ltd.net/. Used by Creative Commons license.

In our studies with SMC and aMCI participants (Manenti et al., 2017, 2020), we applied the experimental protocol used in our previous studies with healthy older adults (Sandrini et al., 2014; Manenti et al., 2016). The procedure included a learning session (Day 1), a reactivation and tDCS session (Day 2, 24 h after Day 1), and two retrieval sessions (Day 3, 48 h after Day 1, and Day 30, 1 month after Day 1). The participants returned to the hospital on Day 3 and 30 without expecting a memory test since when they were contacted for the study, these visits were not described as directly linked with the memory task conducted on Day 1. See Figure 1 for a summary.

#### Day 1—Encoding Session

Twenty short (mean letters: 6.3, SD: 1.0; mean syllables: 2.5, SD: 0.5), concrete (mean concreteness score: 6.3, SD: 0.5) and high frequency (mean frequency score: 24.5, SD: 23.2) words were selected (Barca et al., 2002; Bertinetto et al., 2005).

The experimenter pulled one item (a word printed on piece of card) from a white bag at random and gave it to the participants. The participants were asked to encode each word and then place the card in a blue bag. When all words were placed into the blue bag, the participants were asked to recall as many words as possible. Before the subsequent learning round, the words were placed again in the white bag and mixed. The procedure was repeated five times, and at the end of the learning session, the participants were asked to complete a memory strategies questionnaire (Manenti et al., 2010).

#### Day 2—Reactivation and Transcranial Direct Current Stimulation Session

Twenty-four hours after the learning session, the same experimenter involved on Day 1 in the same experimental room showed the blue bag to the participant and asked if he or she remembered the blue bag and what he or she did with it. The participants were encouraged to describe the procedure but were stopped if they started to recall the words printed on the cards. Previous studies have shown that consolidated memories are automatically reactivated if the participants are in the same experimental room as the learning session (Hupbach et al., 2008; Sandrini et al., 2013). Participants received 15 min of tDCS (active or sham) beginning 10 min after the contextual reminder cue because the reconsolidation process seems to begin about 10 min after memory reactivation (Monfils et al., 2009). During reactivation and tDCS session the participants were encouraged to keep still with open eyes, relax their mind and they were stopped if they started to recall the words printed on the cards.

#### Day 3 and 30—Retrieval Sessions

Forty-eight hours (Day 3) and 1 month (Day 30) after the learning session (Day 1), in the same experimental room and with the same experimenter, the participants were required to recall the words learned during Day 1 (free recall task). When the participants could not remember any more words, an old/new recognition task, which included 20 learned words along with 20 new words, was performed. Target words encoded on Day 1 and both the new words displayed on Day 3 and new words used on Day 30 during the recognition task (which were different from those used on Day 3) were balanced based on variables known to influence memory performance.

### Statistical Analysis

The Gaussian distribution of the four response variables was investigated through graphical inspection and the Shapiro-Wilk test. “Free Recall Day 3” and “Free Recall Day 30” variables showed a right-skewed distribution and were analyzed through generalized linear models. For memory recognition performance, hits minus false alarms rate was computed.

For evaluating the direct effect of predictors on response variables, univariate models (one model for each response variable as the dependent variable) were applied with age, education, CRI total and subscale scores, diagnosis (SMC vs. aMCI), tDCS group (active vs. sham), and encoding performance as single independent variables/predictors. These predictors were previously selected (based on former study evidence), thus inference on them did not need to be adjusted for multiple testing (Rothman, 1990; Proschan and Follmann, 1995).

For Gaussian-distributed variables such as recognition performance (hits—false alarms, both on Day 3 and on Day 30), a linear model was applied, while a generalized linear model (with Tweedie distribution and log link function) was applied for non-Gaussian response variables. The Tweedie distribution is particularly useful and appropriate for modeling data with a cluster of values at zero (as the Free recall variables are).

To evaluate the potential interaction effects of tDCS group and other predictors, multiple models were applied for each of the four response variables as dependent variables and with predictors (same as above), tDCS group, and predictor x tDCS group interaction as independent variables. All models were adjusted for age.

The goodness of fit of the models was assessed through the Akaike Information Criterion (AIC; a lower index indicates a better fit).

Sensations induced by tDCS were compared between the active and the sham groups using Mann-Whitney *U*-test.

Statistical analyses were performed using SPSS (Dell Software, Aliso Viejo, CA, United States). Statistical significance was set at *p* < 0.05.

## Results

### Free Recall

Univariate models showed no effects of tDCS group (sham vs. active) on free recall on Day 3 and on Day 30 (Tables 1, 2), confirming previous results in SMC and aMCI groups (Manenti et al., 2017, 2020). See Supplementary Table 1 for free recall and recognition data.

**TABLE 1:** Free recall—day 3.

Independent variables/predictors	Beta coefficient	*p*-value	AIC
tDCS Group	0.30 (active vs. sham)	0.445	176
Education	0.09	0.069	173.4
CRI total	0.01	0.448	176
CRI education	0.01	0.278	175.5
CRI working activity	0.001	0.941	176.7
CRI leisure time	0.08	0.775	177.3
**Diagnosis**	**1.46** (SMC vs. aMCI)	**<0.001**	**164.7**
**Encoding performance**	**0.20**	**<0.001**	**162.3**
Age	-0.04	0.285	175.5

*tDCS group, sham as reference category; Diagnosis, aMCI as reference category.*

*CRI, Cognitive Reserve Index. Significant results are shown in bold.*

**TABLE 2:** Free recall—day 30.

Independent variables/predictors	Beta coefficient	*p*-value	AIC
tDCS Group	0.12 (active vs. sham)	0.873	130.3
**Education**	**0.14**	**0.048**	126.5
**CRI total**	**0.04**	**0.049**	**126.5**
CRI education	0.01	0.461	129.8
CRI working activity	0.02	0.188	128.6
CRI leisure time	0.02	0.193	128.6
**Diagnosis**	**2.22** (SMC vs. aMCI)	**0.001**	**120.7**
**Encoding performance**	**0.26**	**0.002**	**120.5**
Age	-0.04	0.405	129.6

*tDCS group, sham as reference category; Diagnosis, aMCI as reference category.*

*CRI, Cognitive Reserve Index. Significant results are shown in bold.*

**FIGURE 2:**
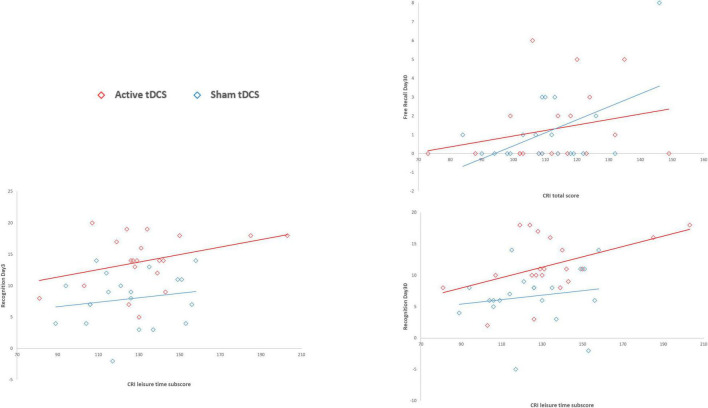
Association between CRI scores and Free Recall and Recognition variables by tDCS group. CRI total score significantly predicted free recall performance only on Day 30, suggesting that higher cognitive reserve led to higher free recall performances only in the long-term follow up (30 days after encoding). The CRI leisure time subscore significantly predicted recognition performance both on Day 3 and on Day 30, suggesting that the higher cognitive reserve, measured as leisure time activities, led to higher recognition performances in both short- and long-term follow-ups (3 and 30 days after encoding).

Diagnosis (SMC vs. aMCI) significantly predicted free recall performance both on Day 3 and on Day 30 (Day 3: β = 1.46, *p* < 0.001, AIC = 164.7; Day 30: β = 2.22, *p* = 0.001, AIC = 120.7), suggesting higher free recall performances in the SMC group than in the aMCI group (both betas > 0). Moreover, the number of words recalled during the last round of the encoding session significantly predicted free recall performance both on Day 3 and on Day 30 (Day 3: β = 0.20, *p* < 0.001, AIC = 162.3; Day 30: β = 0.26, *p* = 0.002, AIC = 120.5), suggesting that greater encoding resulted in higher free recall performances. Finally, education and CRI total scores significantly predicted free recall performance only on Day 30 (education: β = 0.14, *p* = 0.048, AIC = 126.5; CRI total score: β = 0.04, *p* = 0.049, AIC = 126.5), suggesting that higher education and higher cognitive reserve led to higher free recall performances only in the long-term follow up (30 days after encoding). See Figure 2 for details.

For free recall performance on Day 3 and 30, the best predictor was the number of words recalled during the last round of the encoding session (i.e., the encoding performance variable), with an AIC = 162.3 and AIC = 120.5, respectively.

### Old/New Recognition

Univariate models showed that the tDCS group (sham vs. active) significantly predicted recognition performance (hit -false alarms) on Day 3 and on Day 30, indicating higher scores in the active group than in the sham group (Tables 3, 4) and confirming previous results in the SMC and aMCI groups (Manenti et al., 2017, 2020).

**TABLE 3:** Recognition—day 3 (hit-false alarms).

Independent variables/predictors	Beta coefficients	*p*-value	AIC
**tDCS Group**	**6.05** (active vs. sham)	**<0.001**	**234**
Education	0.20	0.359	250
CRI	0.09	0.087	248
CRI education	-0.02	0.640	250.6
CRI working activity	0.05	0.278	249.7
**CRI leisure time**	0.07	**0.029**	246.3
**Diagnosis**	**3.80** (SMC vs. aMCI)	**0.013**	**245.1**
**Encoding performance**	**0.58**	**0.004**	**243.4**
Age	0.06	0.812	250.8

*tDCS group, sham as reference category; Diagnosis, aMCI as reference category.*

*CRI, Cognitive Reserve Index. Significant results are shown in bold.*

**TABLE 4:** Recognition—day 30 (hit-false alarms).

Independent variables/predictors	Beta coefficients	*p*-value	AIC
**tDCS Group**	**4.90** (active vs. sham)	**0.001**	**239.4**
Education	0.29	0.159	248
CRI	0.10	0.055	246.4
CRI education	-0.04	0.430	249.3
CRI working activity	0.07	0.152	247.9
**CRI leisure time**	0.08	**0.011**	243.9
**Diagnosis**	**5.13** (SMC vs. aMCI)	**<0.001**	**238.4**
**Encoding performance**	**0.66**	**0.001**	**239.9**
Age	-0.13	0.407	249.2

*tDCS group, sham as reference category; Diagnosis, aMCI as reference category.*

*CRI, Cognitive Reserve Index. Significant results are shown in bold.*

Diagnosis (SMC vs. aMCI) significantly predicted recognition performance both on Day 3 and on Day 30 (Day 3: β = 3.80, *p* = 0.013, AIC = 245.1; Day 30: β = 5.13, *p* < 0.001, AIC = 238.4), suggesting higher performances in the SMC group than in the aMCI group. Moreover, the number of words recalled during the last round of the encoding session significantly predicted recognition performance both on Day 3 and on Day 30 (Day 3: β = 0.58, *p* = 0.004, AIC = 243.4; Day 30: β = 0.66, *p* = 0.001, AIC = 239.9), suggesting that greater encoding resulted in higher recognition performances. Finally, the CRI leisure time subscores significantly predicted recognition performance both on Day 3 and on Day 30 (Day 3: β = 0.07, *p* = 0.029, AIC = 246; Day 30: β = 0.08, *p* = 0.011, AIC = 243.9), suggesting that the higher cognitive reserve, measured as leisure time activities, led to higher recognition performances in both short- and long-term follow-ups (3 and 30 days after encoding). See Figure 2 for details.

The best predictors of recognition on Days 3 and 30 were the tDCS group (AIC = 234) and diagnosis (AIC = 238), respectively.

**FIGURE 3:**
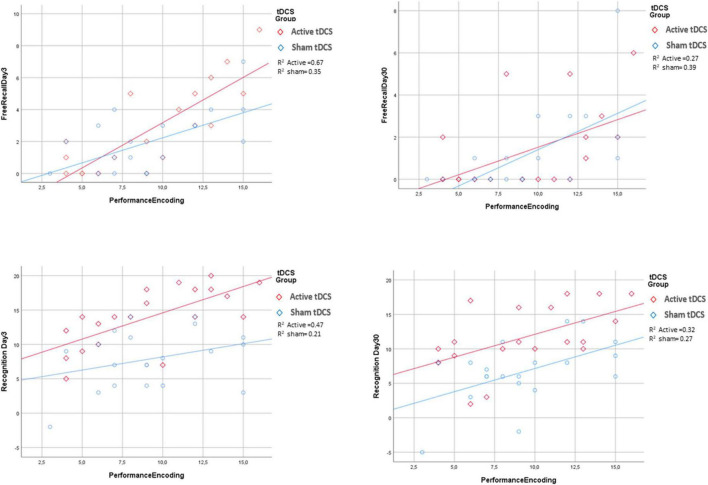
Association between encoding performance and the four response variables by tDCS group. For all the response variables, increasing of performance encoding was associated with an increase of the response variable irrespectively for tDCS groups. *R*^2^ is goodness of fit index of the two regression lines fitted for active and sham tDCS groups separately.

**FIGURE 4:**
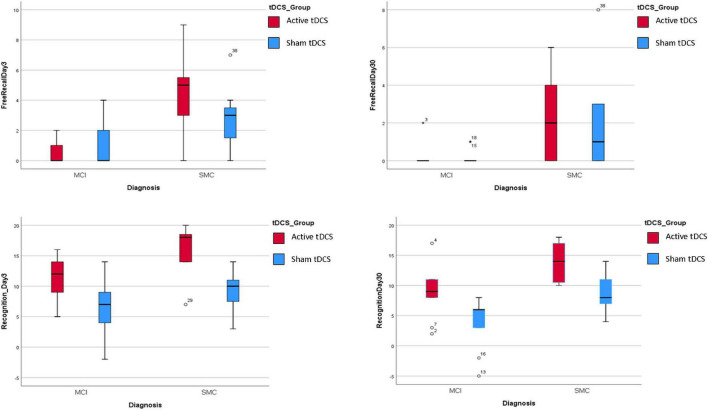
Association between diagnosis and the four response variables by tDCS group. For all the response variables, in the Active treatment the scores for all the 4 response variables were higher than in the sham treatment irrespectively for Diagnosis groups.

Finally, multiple models were performed to evaluate the potential interaction effect of tDCS group x predictors in explaining the four response variables (Supplementary Tables 2–5). None of the interaction terms were found to be significant, which indicated that the effect of the predictors on response variables was apparent irrespective of tDCS treatment (sham or active) group (see Figure 3). Similar outcomes were obtained for the diagnosis x tDCS treatment interaction (Figure 4).

### Transcranial Direct Current Stimulation Sensations

The tDCS sensations scores reported by the active and sham groups were similar (SMC: Active tDCS group: 1.09, SD 0.7, Sham tDCS group: 1.45, SD 0.8; *p* = 0.27; aMCI: Active tDCS group: 1.4, SD 1.1, Sham tDCS group: 1.0, SD 0.9; *p* = 0.38). Hence, there are no reasons to reject the blinded character of this study on the basis of these results.

## Discussion

In the present study, we explored how the magnitude of tDCS-induced reconsolidation effects might be directly and/or indirectly modulated by factors such as cognitive reserve, education level, diagnosis and encoding performance. This aim was investigated by analyzing free recall and recognition performances on Day 3 and at Day 30 as dependent variables and tDCS group, cognitive reserve, education level, diagnosis and encoding performance as predictors.

In agreement with our preview results (Manenti et al., 2017, 2020) the tDCS group (active vs. sham) significantly predicted recognition memory but not free recall, as evidenced by higher recognition scores in the active tDCS group than in the sham group. In detail, beta coefficients of active vs. sham were positive 6.05 and 4.90 (and significantly different from zero: *p* < 0.001 and *p* = 0.001) for both Recognition on Day 3 and 30, respectively (Tables 3, 4). This indicates that to be in Active tDCS group significantly predicts an increase of Recognition of 6.05 on Day 3 and of 4.9 on Day 30 with respect to be in Sham group. For free recall these coefficients were lowest and not significantly different from zero indicating that there was not difference in active vs. sham tDCS group for the free recall score. The fact that we did not find a significant association between tDCS group and free recall performance is in line with other studies that reported severe deficits in free recall in SMC and aMCI (Hertzog et al., 2000; Dubois and Albert, 2004; Bennett et al., 2006; Hohman et al., 2011; Talamonti et al., 2020).

Encoding performance predicted memory retrieval performance, such that more words recalled during the last round of the encoding session was associated with better retrieval performance.

Diagnosis (SMC vs. aMCI) also predicted memory retrieval performance, which suggested higher performances in the SMC group than in the aMCI group. These findings suggested that retrieval performance is different between SMC and aMCI subjects.

This study also investigated whether memory retrieval performance was associated with protective factors such as cognitive reserve. Our main finding was that higher CRI leisure time subscores predicted better retrieval performances on Days 3 and 30.

These results are consistent with a large body of evidence that suggests that engagement in leisure and social activities may also be protective against cognitive deterioration (Hughes et al., 2013; Fallahpour et al., 2016; Petkus and Gomez, 2021). Engagement in social leisure activities has been previously identified as a factor that provide older adults maintain their cognitive status (Barnes et al., 2004; Haslam et al., 2014; Bourassa et al., 2017; Hwang et al., 2018; Fratiglioni et al., 2020) and preventing or delaying further cognitive decline in MCI (Fratiglioni et al., 2004; Hughes et al., 2013).

Our findings are in accordance with the recent literature that suggest the importance of developing multifactorial interventions to slow cognitive decline and delay the onset of dementia (Stephen et al., 2021). Instead, we did not find a relationship between the working activity subscore of the CRI and memory performance, and this result may reflect the fact that the subjects participating in the study were retired and had ended their professional activity long before the study was conducted.

Importantly, cognitive reserve, education level, diagnosis and encoding performance did not directly and/or indirectly modulate memory enhancement effects induced by tDCS because the results did not show any tDCS group × predictor interaction effects, which indicated that the effects of the predictors on retrieval performance occurred irrespective of tDCS group (active vs. sham).

Some limitations of the current study should be acknowledged. First, the findings reported in the present manuscript should be reproduced in larger SMC and aMCI samples before firm conclusions can be drawn because relatively small groups were recruited. Larger cohorts might help to better investigate intergroup and intragroup variability both on Day 1 performance and in the demographic and clinical characteristics that could influence the experimental memory performance that was measured. Moreover, we did not include a control stimulation site, and accordingly, non-specific effects of the stimulation cannot be ruled out.

Although this study sheds light on predicting factors of episodic memory retrieval in this reconsolidation paradigm in individuals with SMC and aMCI, future studies will need to determine the factors that destabilize memories and make them vulnerable to modifications through reconsolidation in the aging population. In addition, it is important to determine whether the effect of tDCS applied during reconsolidation could be useful in populations with SMCs or clinically significant memory impairments by enhancing the ability to recall information and improve daily life activities. The application of multiple sessions of tDCS during reconsolidation might be beneficial in determining long-lasting positive effects of this non-invasive intervention on memory deficits.

Although neuroimaging studies have begun to identify brain networks associated with memory reconsolidation (Gais et al., 2007; Schwabe et al., 2014; Forcato et al., 2016; Bavassi et al., 2019), it is important to better understand the brain mechanisms associated with this memory process. This knowledge will help develop effective interventions that strengthen existing memory through reconsolidation in populations with memory decline.

In conclusion, tDCS group, diagnosis, encoding performance, and leisure time activities are predictive factors of delayed memory retrieval in this reconsolidation paradigm in populations with an increased risk of developing AD.

## Data Availability Statement

The datasets presented in this study can be found in online repositories. The names of the repository/repositories and accession number(s) can be found below: Mendeley Data, V1, doi: 10.17632/rfm32symvh.1.

## Ethics Statement

The studies involving human participants were reviewed and approved by the Ethics committee of the Istituto di Ricovero e Cura a Carattere Scientifico (IRCCS) Centro San Giovanni di Dio Fatebenefratelli, Brescia, Italy. The patients/participants provided their written informed consent to participate in this study.

## Author Contributions

MC, CF, EG, RM, and MS: conception and methodology. MC, CF, and MS: data curation. MC, CF, EG, GB, RM, and MS: writing—original draft preparation and writing—review and editing. All authors have read and agreed to the published version of the manuscript.

## Conflict of Interest

The authors declare that the research was conducted in the absence of any commercial or financial relationships that could be construed as a potential conflict of interest.

## Publisher’s Note

All claims expressed in this article are solely those of the authors and do not necessarily represent those of their affiliated organizations, or those of the publisher, the editors and the reviewers. Any product that may be evaluated in this article, or claim that may be made by its manufacturer, is not guaranteed or endorsed by the publisher.
