# Supplementary material for: tDCS-Induced Memory Reconsolidation Effects: Analysis of Prominent Predicting Factors
Source: Front Neurosci. 2022 Mar 17;16:814003. doi: 10.3389/fnins.2022.814003 (PMC8969754; doi:10.3389/fnins.2022.814003)
Supplement: Supplementary file 1 [file Table_1.docx]

Supplementary Results

**Table S1 Free recall and Recognition data**

|  |  |  | **Free Recall** | | **Recognition** | |
| --- | --- | --- | --- | --- | --- | --- |
|  |  |  | **Day 3** | **Day 30** | **Day 3** | **Day 30** |
| **MCI** | **Active tDCS** | **Mean** | 3,33 | 1,11 | 11,22 | 8,78 |
|  |  | **SD** | 4,33 | 3,33 | 3,29 | 4,21 |
|  | **Sham tDCS** | **Mean** | 5,00 | 1,11 | 6,44 | 3,78 |
|  |  | **SD** | 7,07 | 2,20 | 4,60 | 4,16 |
| **SMC** | **Active tDCS** | **Mean** | 21,82 | 10,91 | 16,18 | 13,82 |
|  |  | **SD** | 12,30 | 10,83 | 3,56 | 3,33 |
|  | **Sham tDCS** | **Mean** | 14,09 | 9,55 | 9,09 | 9,00 |
|  |  | **SD** | 9,00 | 11,37 | 3,26 | 3,07 |

Multiple generalized linear models

**Table S2 Free recall - day 3**

| **Independent variables/predictors** | Beta coefficient | p-value | AIC |
| --- | --- | --- | --- |
| Age  Education  tDCS group  Education * tDCS group | -0.12  0.07  0.19 (active vs. sham)  0.02 (active vs. sham) | 0.781  0.166  0.858  0.858 | 178.6 |
| Age  CRI  tDCS group  CRI* tDCS group | -0.04  0.01  0.47 (active vs. sham)  0.001 (active vs. sham) | 0.232  0.574  0.886  0.995 | 180.1 |
| Age  CRI-education  tDCS group  CRI-education * tDCS group | -0.03  0.003  -1.09 (active vs. sham)  0.02 (active vs. sham) | 0.391  0.418  0.663  0.545 | 179.5 |
| Age  CRI working activity  tDCS group  CRI working activity * tDCS group | -0.04  0.01  2.19 (active vs. sham)  -0.02 (active vs. sham) | 0.206  0.936  0.401  0.491 | 180 |
| Age  CRI leisure time  tDCS group  CRI leisure time* tDCS group | -0.05  0.003  0.40 (active vs. sham)  -0.0001 (active vs. sham) | 0.192  0.701  0.865  0.999 | 180.3 |
| Age  **Diagnosis**  tDCS group  Diagnosis* tDCS group | -0.03  0.94 (SMC vs. aMCI)  -0.36 (active vs. sham)  0.92 (active SMC vs.  active aMCI vs. sham SMC) | 0.281  **<0.001**  0.804  0.249 | 168.2 |
| Age  **Encoding performance** tDCS group  Encoding performance * tDCS group | 0.01  0.15  -0.75 (active vs. sham)  0.09 (active vs. sham) | 0.881  **<0.001**  0.489  0.371 | 167.3 |

Significant results are shown in bold. CRI: Cognitive Reserve Index.

The colored lines indicate models for CRI (blue) and corresponding CRI subscale (light blue)

**Table S3. Free recall - Day 30**

| **Independent variables/predictors** | Beta coefficient | p-value | AIC |
| --- | --- | --- | --- |
| Age  Education  tDCS group  Education* tDCS group | 0.01  0.14  -0.04 (active vs. sham)  0.02 (active vs. sham) | 0.912  0.085  0.983  0.880 | 132.3 |
| Age  CRI  tDCS group  CRI* tDCS group | -0.04  0.04  1.49 (active vs. sham)  -0.01 (active vs. sham) | 0.458  0.062  0.742  0.785 | 131.7 |
| Age  CRI-education  tDCS group  CRI-education* tDCS group | -0.40  0.01  -0.05 (active vs. sham)  0.004 (active vs. sham) | 0.461  0.627  0.988  0.911 | 135.1 |
| Age  **CRI working activity**  tDCS group  CRI working activity* tDCS group | -0.03  0.07  6.86 (active vs. sham)  -0.06 (active vs. sham) | 0.534  **0.039**  0.087  0.106 | 131.3 |
| Age  CRI leisure time  tDCS group  CRI leisure time* tDCS group | -0.06  0.03  1.69 (active vs. sham)  -0.01 (active vs. sham) | 0.252  0.116  0.632  0.679 | 132.9 |
| Age  **Diagnosis**  tDCS group  Diagnosis* tDCS group | -0.03  2.07 (SMC vs. aMCI)  0.11 (active vs. sham)  0.15 (active SMC vs.  active aMCI vs. sham SMC) | 0.609  **0.002**  0.805  0.915 | 126.4 |
| Age  **Encoding performance**  tDCS group  Encoding performance * tDCS group | 0.03  0.37  1.77 (active vs. sham)  -0.15 (active vs. sham) | 0.583  **0.001**  0.357  0.363 | 125.1 |

Significant results are shown in bold. CRI: Cognitive Reserve Index.

The colored lines indicate models for CRI (blue) and corresponding CRI subscale (light blue)

**Table S4. Recognition - Day 3 (hit-false alarms)**

| **Independent variables/predictors** | Beta coefficient | p-value | AIC |
| --- | --- | --- | --- |
| Age  Education  tDCS group  Education* tDCS group | -0.05  0.16  5.22 (active vs. sham)  0.11 (active vs. sham) | 0.734  0.261  0.129  0.749 | 237.8 |
| Age  CRI  tDCS group  CRI* tDCS group | -0.12  0.05  0.23 (active vs. sham)  0.05 (active vs. sham) | 0.341  0.087  0.980  0.524 | 235.5 |
| Age  CRI-education  tDCS group  CRI-education* tDCS group | -0.14  -0.03  3.91 (active vs. sham)  0.02 (active vs. sham) | 0.307  0.686  0.628  0.771 | 238.8 |
| Age  CRI working activity  tDCS group  CRI working activity* tDCS group | -0.10  0.09  11.11 (active vs. sham)  -0.05 (active vs. sham) | 0.437  0.117  0.192  0.568 | 236.6 |
| Age  CRI leisure time  tDCS group  CRI leisure time* tDCS group | -0.19  0.03  1.46 (active vs. sham)  0.03 (active vs. sham) | 0.143  0.055  0.839  0.535 | 234.6 |
| Age  **Diagnosis**  **tDCS group**  Diagnosis* tDCS group | -0.12  2.38 (SMC vs. aMCI)  4.78 (active vs. sham)  2.65 (active SMC vs.  active aMCI vs. sham SMC) | 0.300  **0.001**  **<0.001**  0.256 | 228.9 |
| Age  **Encoding performance**  tDCS group  Encoding performance * tDCS group | -0.03  0.37  2.55 (active vs. sham)  0.39 (active vs. sham) | 0.772  **<0.001**  0.401  0.205 | 226 |

Significant results are shown in bold. CRI: Cognitive Reserve Index.

The colored lines indicate models for CRI (blue) and corresponding CRI subscale (light blue)

**Table S5. Recognition - Day 30 (hit-false alarms)**

| **Independent variables/predictors** | Beta coefficient | p-value | AIC |
| --- | --- | --- | --- |
| Age  Education  tDCS group  Education* tDCS group | -0.14  0.32  6.53 (active vs. sham)  -0.15 (active vs. sham) | 0.384  0.224  0.070  0.690 | 241.3 |
| Age  CRI  tDCS group  CRI* tDCS group | -0.19  0.07  2.90 (active vs. sham)  0.02 (active vs. sham) | 0.142  0.058  0.767  0.824 | 239.5 |
| Age  CRI-education  tDCS group  CRI-education* tDCS group | -0.25  -0.02  9.86 (active vs. sham)  -0.05 (active vs. sham) | 0.077  0.359  0.240  0.570 | 242 |
| Age  **CRI working activity**  tDCS group  CRI working activity* tDCS group | -0.17  0.16  17.08 (active vs. sham)  -0.12 (active vs. sham) | 0.203  **0.036**  0.051  0.172 | 238.7 |
| Age  **CRI leisure time**  tDCS group  CRI leisure time* tDCS group | -0.28  0.04  -3.35 (active vs. sham)  0.06 (active vs. sham) | **0.027**  **0.016**  0.645  0.262 | 235.5 |
| Age  **Diagnosis**  **tDCS group**  Diagnosis* tDCS group | -0.17  4.84 (SMC vs. aMCI)  5.00 (active vs. sham)  0.30 (active SMC vs.  active aMCI vs. sham SMC) | 0.136  **<0.001**  **<0.001**  0.896 | 227.5 |
| Age  **Encoding performance**  tDCS group  Encoding performance * tDCS group | -0.08  0.62  4.92 (active vs. sham)  0.02 (active vs. sham) | 0.500  **<0.001**  0.128  0.956 | 231 |

Significant results are shown in bold. CRI: Cognitive Reserve Index.

The colored lines indicate models for CRI (blue) and corresponding CRI subscale (light blue)
